# Letter from the Editor in Chief

**DOI:** 10.19102/icrm.2020.110101

**Published:** 2020-01-15

**Authors:** Moussa Mansour


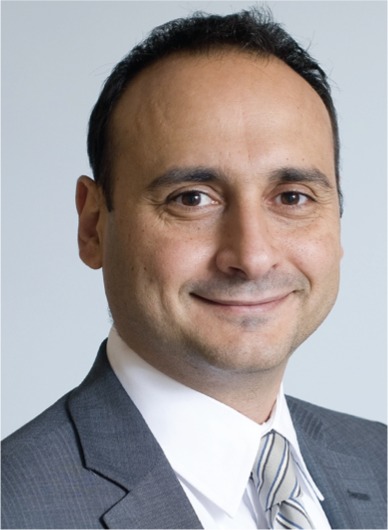


Dear Readers,

As we get ready to greet 2020, I would like to explore some of the major advancements in the field of cardiac electrophysiology that we witnessed in the past year and ponder where we may be heading from here.

First, driven by the results of landmark clinical studies such as CASTLE AF and CABANA and improvements in procedural efficacy and technology, catheter ablation for atrial fibrillation (AF) grew significantly. A particularly beneficial practice that gained prevalence last year is the delivery of high-power, short-duration energy, which reduces both the negative effects of catheter instability on lesion quality and the overall duration of the procedure. Novel catheters containing elaborate temperature sensors to help avoid steam pop when high power is used were also introduced; two such catheters (QDot Micro™ from Johnson & Johnson, New Brunswick, NJ, USA and DiamondTemp™ from EPIX Therapeutics, Santa Clara, CA, USA) may become commercially available in 2020. Radiofrequency balloon catheters, while still investigational, were also examined in 2019, and the STELLAR clinical study is expected to be completed in 2020. An investigational expandable lattice electrode ablation catheter was similarly introduced in 2019 and is expected to undergo multicenter clinical studies in 2020. Finally, pulsed-field ablation, also called irreversible electroporation, is gathering significant momentum; at least two clinical trials using this novel energy source will be conducted in 2020.

Regarding the ablation of persistent AF, three multicenter clinical studies (PRECEPT, PERSIST-END, and STOP Persistent AF) were completed in 2019, with the results possibly forthcoming in 2020. Further, a trend toward adopting anatomically guided lesion sets involving the posterior left atrium, the vein of Marshall, and the coronary sinus appears to be gaining popularity, with clinical studies expected in 2020.

In the field of left atrial appendage (LAA) closure, enrollment in two studies (WATCHMAN FLX and AMPLATZER Amulet LAA Occluder) to examine the safety and effectiveness of two new devices was completed in 2019, while a third such device is under investigation in the ongoing WAVECREST multicenter study. Separately, clinical studies seeking new indications for LAA closure such as ASAP TOO, OPTION, and WATCH TAVR are expected to continue enrollment through 2020.

Finally, His-bundle pacing, leadless single-/dual-chamber pacing, and subcutaneous/substernal defibrillation are expected to grow significantly in 2020.

In summary, 2019 was a prolific year of growth and innovation for the field and this trend is expected to continue in 2020. At *The Journal of Innovations in Cardiac Rhythm Management*, we continue to work hard to bring to you new information in this field, focusing specifically on efficacy in clinical practice.

On behalf of the editorial team and editorial board, I wish you a happy, healthy, and prosperous new year.

Sincerely,


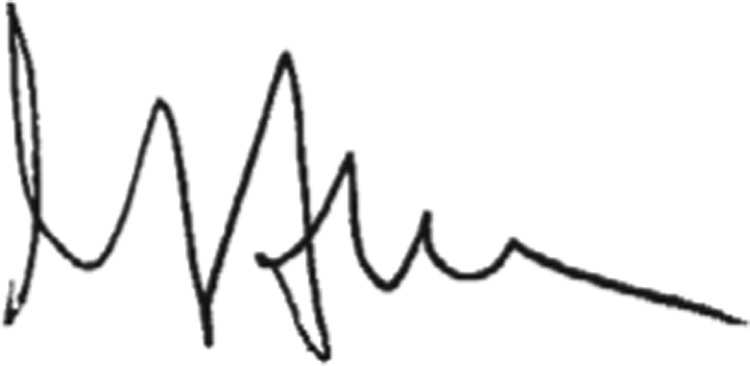


Moussa Mansour, MD, FHRS, FACC

Editor in Chief

The Journal of Innovations in Cardiac Rhythm Management

MMansour@InnovationsInCRM.com

Director, Atrial Fibrillation Program

Jeremy Ruskin and Dan Starks Endowed Chair in Cardiology

Massachusetts General Hospital

Boston, MA 02114

